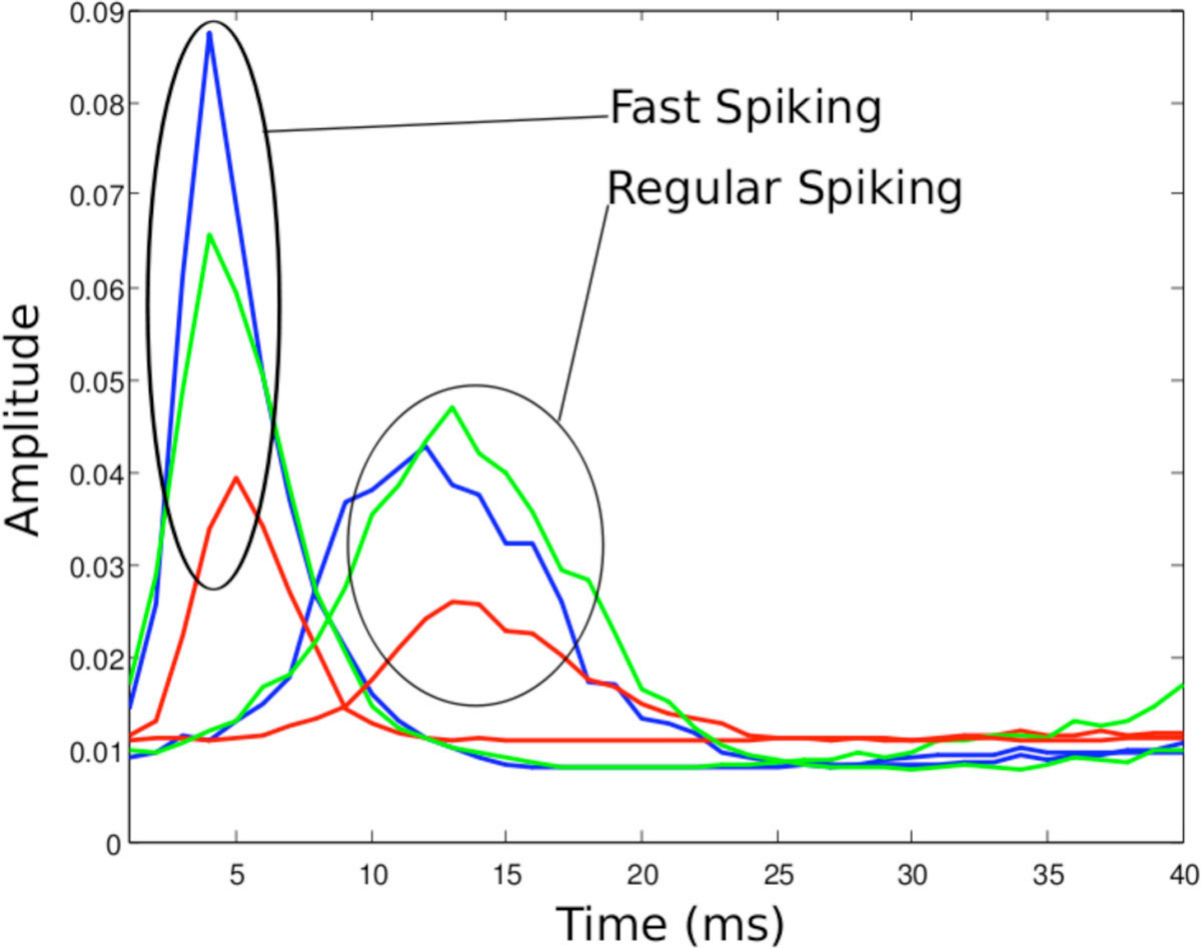# Self-tuning spike-timing dependent plasticity curves to simplify models and improve learning

**DOI:** 10.1186/1471-2202-14-S1-P189

**Published:** 2013-07-08

**Authors:** Micah Richert, Botond Szatmary, Eugene Izhikevich

**Affiliations:** 1 Brain Corporation, San Diego, CA, USA

## 

Plasticity for feed-forward excitation ought to optimally assign credit to which synapses cause a postsynaptic cell to spike. It is common to use a double-exponential fit of the LTP and LTD curves [1]; however, exponential curves are not always optimal and are prone to some pathologies. For example, if there are repeated patterns in the input spikes, learning will degenerate to only the selection of the earliest spikes [2]. Often the parameters for STDP are hand tuned for particular problems and networks.

Measuring of the cross-correlogram offline can provide useful insight as to what the optimal STDP curve should be. We propose an adaptive STDP curve that is derived online from the cross-correlogram, and will discuss its relationship to biology. This dynamic plasticity automatically incorporates an estimate of the dendritic and neuronal integration/processing time in order for a presynaptic input to cause a postsynaptic spike. This plasticity results in faster learning and greater diversity in a model of V1 simple cells. Further, for different neural models and input statistics, different STDP curves will be learned and yet still result in good V1 receptive fields. Because the STDP curve is adaptive to the statistics for each cell, it can be different for each cell in the same population. The model requires only a few meta parameters, which are intuitive, and learning is stable over a large range. Most importantly, instead of having to fiddle with parameters, this synapse model is self-tuning.

For a model of V1 which uses two feed-forward populations with two different neural dynamics ([3]: Regular Spiking and Fast Spiking) the dynamic plasticity finds significantly different STDP curves and even for neurons with the same neural dynamics slightly different curves are learned (Figure 1). While the peak of the curves are approximately the same, there can be 1-2ms jitter in the peaks which have physiological consequences: neurons with later STDP curves generally have larger receptive fields. Also, it should be noted that none of the LTP curves are significantly higher than baseline at t = 1; this is because unless synaptic weights are unrealistically large, no pre-spike can cause a post-spike within 1ms. This difference between the normal exponential fit of the LTP curve [1] and our dynamic LTP at the beginning of curve (t near 1) is what contributes to better assigning cause to which synapses contributed to a spike. Because of this better credit-assignment, the model is able to learn V1 receptive fields faster than a model using hand-tuned curves. When hand-tuning curves, often different plasticity rules are required for each neural population or even if some other properties of the network change, such as the level of inhibition. This dynamic rule automatically finds an appropriate curve for any neural model.

**Figure 1:**